# Belonging as a part of the pathway to working life: immigrant women’s experiences in Sweden

**DOI:** 10.1186/s12889-026-29688-w

**Published:** 2026-10-05

**Authors:** Marie Lydell, Cecilia Garell, Eva-Carin Lindgren, Marie-Helene Zimmerman Nilsson, Krister Hertting

**Affiliations:** 1 School of Health and Welfare, Halmstad University, Box 823, Halmstad, 301 18 Sweden; 2 Division of Educational Science and Languages, Department of Social and Behavioural Studies, University West, Trollhättan, Sweden

**Keywords:** Belonging, Integration, Women, Working life

## Abstract

**Background:**

Coming to a new society involves complex processes of incorporating change into immigrants’ lives. For many immigrant women, participating in working life is a crucial period of transition into a new society, offering opportunities for improved life. *Objective*: Therefore, this study aims to explore the pathways to working life experienced by immigrant women in Sweden, with a particular focus on the experience of belonging.

**Method:**

A total of 11 immigrant women from five different non-European countries were interviewed. The interviews were transcribed verbatim and analysed using reflexive thematic analysis. The concepts of cultural, relational, sensory and temporal belonging were used in the analysis to interpret experiences of pathways to working life.

**Results:**

Overall, the immigrant women described the pathway into the working life as a central part of their integration process. However, it was often complex and challenging. Experiences of cultural belonging included learning the Swedish language and understanding the workplace culture. Relational belonging, in the form of giving and receiving support and forming social networks, played a crucial role in shaping one’s sense of belonging. Sensory belonging was about feeling accepted and being a valued member of the workplace. Finally, temporal belonging involved the woman reflecting on a journey spanning time, showing a clear sense of progression toward a future goal.

**Conclusion:**

To include and support immigrant women throughout their pathways to working life requires workplaces to enhance cultural, relational, sensory and temporal belonging. Such measures can promote the possibilities of integration in working life.

**Supplementary Information:**

The online version contains supplementary material available at https://doi.org/10.1186/s12889-026-29688-w.

## Introduction

Global forced displacement has increased sharply over the past decade, rising from approximately 40 million people in 2012 to 108.4 million in 2022, of whom 45.9 million were displaced across borders [1]. Refugee numbers continued to grow in early 2023, alongside a more rapid increase in asylum seekers, while a majority of those in need of international protection originate from Syria, Ukraine, and Afghanistan. These global trends are reflected nationally, as Sweden’s foreign-born population has expanded steadily; in 2021, around two million residents were born abroad, representing approximately 20% of the population [2].

However, today the government in Sweden describes a shift towards stricter migration and integration requirements, including greater emphasis on language, self-sufficiency, and citizenship, as well as reduced asylum-related immigration [3].

Entering the working life of the new society and culture is crucial for economic self-sufficiency, building social networks, learning the language and cultural norms, restoring self-esteem, and fostering a sense of inclusion and belonging [4–8]. However, the integration of refugees into Swedish working life is relatively slow [9]. For the general refugee population, the average time to first employment after arrival in Sweden was 3 years [10], although research shows that employment plays an important role in refugees’ integration into society and their health [11]. After 15 years in Sweden, only 59% of refugee women were established in the Swedish working life, compared to 82% of native women [9]. Hence, in Sweden, the least represented group of immigrant women in the working life is women with refugee backgrounds [2].

Women with refugee backgrounds, in general, are facing a higher probability of experiencing longer periods of inactivity in the educational system and working life compared to refugee men, leading to a more challenging pathway to working life integration in a new society [10]. Moreover, at work, immigrant women risk facing double discrimination due to both their ethnicity and gender, which can be expressed as exclusion from workplace networks, disadvantages in occupational status, fewer possibilities for job promotion, and lower salaries [12]. The challenge of acquiring employment risks negatively affecting health and feelings of agency and belonging in the new society [8].

Studies have identified both barriers [8, 13, 14] and facilitators [15, 16] for immigrant women entering the working life in their new country. Possessing a higher education degree increases the probability of a more rapid transition to working life, even though it does not guarantee employment stability [10]. Dimitriadis [17] and van Dijk et al. [6] argue that engaging in language learning and vocational training can facilitate the integration and settlement processes in the host society. Immigrants’ strategies for entering the working life, as described by Kosny et al. [18], included volunteer work to expand networks and enhance cultural learning, participating in employment programs offered by settlement organisations, utilising social networks, and accepting low-quality survival jobs to gain experience in the host society and achieve financial solvency. In particular, developing social networks, both within refugee and local communities, improved the chances of integrating into the new society and the working life [4, 12, 18–20].

Structures and organisations surrounding working life, such as civil society communities, can provide access to valuable relationships with people in the new country [21, 22], promoting health and a sense of belonging [23, 24]. Having strong social networks facilitates a variety of positive outcomes, such as reducing stress and protecting refugees’ self-esteem during experiences of discrimination [25]. Nyström et al. [21] emphasise that civil society, offering meaningful activities, is a crucial complement to education and public services. In the long run, this leads to better opportunities for entry into working life [18]. On the other hand, many refugees feel left on their own and need more help with job seeking, which can affect their health [26].

When refugees enter and participate in working life, social support from managers and colleagues, language learning, and a positive workplace culture all contribute to health and a sense of belonging, which, in turn, positively impact the work environment and job satisfaction [12, 15]. Another important aspect for a sense of belonging was social cohesion with both other immigrants and natives at the workplace [12, 19]. According to Cobb et al. [25], social cohesion between immigrants and natives can be explained by the notion that immigrants develop dual cultural identities encompassing both their culture of origin and the host country’s. They often report greater psychological well-being, higher life satisfaction, and greater flexibility in meeting demands within their social context than refugees who use other acculturation strategies.

Recent scholarship has called for a reorientation of integration research towards its constructive dimensions, emphasising how social relations, agency, and belonging shape refugees’ transitions into new societal contexts [19, 25]. Despite this shift, immigrant women in Sweden continue to face structural constraints in establishing sustainable pathways into working life, reflecting persistent gendered inequalities in labour market access and integration outcomes [10]. This discrepancy underscores the need for studies that examine not only barriers to labour market entry but also the relational and meaning-making processes through which immigrant women navigate work and societal inclusion. Taken together, existing research points to an urgent need to deepen understanding of how immigrant women in Sweden actively construct attainable pathways into working life and how these processes are inseparable from experiences of belonging within a new social and institutional context [10, 12, 23]. Building on this body of research, the study aims to explore how immigrant women in Sweden construct and navigate pathways into working life, with particular attention to the role of belonging. We specifically aim to answer the following research questions: (1) How do immigrant women in Sweden describe and experience their pathways into working life? (2) How do immigrant women in Sweden describe and experience their workplace? Drawing on belonging as an analytical lens, we examine recognition, relational positioning, and social inclusion in relation to institutional and structural conditions shaping participation in working life and broader societal integration.

### Theoretical framework

By shifting the focus from integration to belonging, aspects such as relationality, agency, and everyday existence come into the spotlight, which are essential for working life [5, 6]. Belonging is more than just having employment; it is about feeling “at ease” in a social environment [27, 28]. Belonging is a dynamic, relational construct formed through interactions, emotions, and power dynamics [29, 30]. As such, belonging provides an opportunity to explore how people navigate the boundaries of inclusion and exclusion in their daily lives.

Belonging can be understood as a multidimensional, continually negotiated process through which immigrant women position themselves within cultural, relational, sensory, and temporal contexts during the course of integration and participation in the working life. Building on May’s [27, 28] conceptualisation, cultural belonging is shaped by the interaction between women’s internalised cultural frameworks and the dominant cultural norms of the receiving society, where boundaries of “accepted” behaviour, values, and identities influence who is perceived as eligible to belong in workplaces and broader social spheres. Relational belonging emerges as immigrant women navigate social interactions in employment settings and everyday life, encountering expectations, recognition, and categorisation processes that delineate opportunities for participation and advancement. These relations, often structured by gendered and racialised norms, condition experiences of inclusion and exclusion and reflect the dynamic interplay between individual trajectories and wider social change [27, 28]. Hence, the cultural and relational aspects of belonging are framed by what Yuval-Davis [31] refers to as the politics of belonging, which argues that power relations and boundary-making processes determine who is included in or excluded from a community or nation. Finally, May [28] elucidates the sense of belonging, highlighting how embodied engagements with new environments, such as workplaces, neighbourhoods, and public institutions, shape emotional attachments, comfort, and familiarity, which, in turn, influence women’s sense of competence and ease in their daily working lives. The temporal self [32] underscores that the integration processes of immigrant women unfold through the coexistence of past experiences, present constraints, and imagined futures, meaning that belonging develops and changes over time. Altogether, these dimensions illuminate how belonging for immigrant women in working-life contexts is an ongoing, reflexive process shaped by structural conditions, gendered expectations, and personal histories.

Therefore, we argue that belonging offers a more analytically powerful lens than integration, as it captures how immigrant women’s pathways into working life are shaped by relational, embodied, and temporal negotiations rather than solely by working life participation [27, 28]. As a dynamic process structured by power and boundary-making, belonging reveals how inclusion and exclusion in working life are continuously produced at the intersection of social relations, cultural norms, and personal trajectories [29, 31].

## Method

### Design

The study was grounded in a narrative research design, which focuses on understanding individuals’ experiences through the stories they share. It examines how people construct meaning from events in their lives, emphasising the sequence, context, and interpretation of these experiences as expressed in their own words [33].

### Participants

Purposeful sampling was used to identify and select information-rich participants whose experiences were particularly relevant to the study’s research aim. The sample, therefore, comprised eleven immigrant women with experiences of pathways into working life in Sweden [34]. The study’s inclusion criteria were limited to immigrant women from non-European countries who had work experience in Sweden and could understand and speak Swedish. The second author made contact with responsible persons at a local church (Swedish Church), the local school for Swedish for Immigrants (SFI), and during a meeting with Women On Wednesdays, an organisation providing networking opportunities for foreign women [35] to get access to volunteers for interviews. These contact persons received information about the project and were then able to provide contact details to immigrant women interested in participating in the study. The immigrant women were between 22 and 44 years old and had lived in Sweden for 3 to 10 years at the time of the interviews. They were from different parts of Sweden, mainly western Sweden, but also southern and northern Sweden. We also wanted all of them to have employment. See Table 1 for the participants’ characteristics. The immigrant women were contacted and informed about the research project’s outline, the study’s aim, how the collected data would be handled, and the confidentiality procedures.

**Table 1:** Characteristics of the participants

Home country	Afghanistan (1)
	Kazakhstan (1)
	Iran (1)
	Syria (7)
	Yemen (1)
Year born	1978–2002 (median 1989)
Years in Sweden	3–10 years (median 7 years)
Reason for migration	Accompanied by partner (2)
	Refugee (8)
	Unaccompanied youth (1)
Marital status	Living together with partner (2)
	Married (7)
	Single (2)
Highest education	University (9)
	Upper secondary school (2)
Employment	Care assistant
	Economist
	Architect
	Doctor
	Restaurant personnel
	Teacher
	Assistant at an IT company
	Customer receptionist
	Project manager
	Group manager
	Nurse
Years in current work	<1–6 years (median 1 year)

### Data production and procedure

The empirical material was generated through individual semi-structured interviews designed to elicit the life stories of immigrant women. The participants chose the interview location. Most interviews were conducted in a quiet room at the university, as suggested by the interviewer; others took place in public libraries; one was conducted in a participant’s home; and some (*n* = 5) were conducted via Zoom. At the start of each interview, participants completed a brief background questionnaire, which provided the information presented in Table 1. An interview guide was used, and follow-up questions were used to deepen the interview when needed (supplementary file). The interview guide focused on experiences of pathways into working life. The life story itself was central, and the second author’s primary role during the interviews was to listen. In face-to-face interviews, a 15–20-minute coffee break was included to facilitate informal conversation and ease interaction.

At the end of each interview, participants were invited to add any further reflections. All interviews were audio-recorded and lasted between 60 and 130 min (median 100 min), except for one case in which the participant declined to be recorded. As the interview was not audio-recorded, the participant’s responses were documented in detail through written notes taken throughout the interview. All recordings were transcribed verbatim.

### Data analysis

The analysis followed an abductive logic, moving iteratively between the interview narratives and relevant theoretical concepts, without using any AI-assistants. While the reflexive thematic analysis [36] was grounded in the women’s stories, theoretical perspectives on belonging informed the interpretation and refinement of themes. Within reflexive thematic analysis, such flexible and recursive engagement with data is consistent with the methodological and epistemological openness emphasised by Braun and Clarke [36].

The iterative engagement was integrated into the different analytic phases in the following steps: During the first step, repeated readings of the narratives were done by all authors, experienced researchers in qualitative methods, initial impressions were allowed to emerge inductively, while preliminary theoretical sensitising concepts (e.g., integration, inclusion, pathways) informed reflections on possible meanings without determining them. Steps 2 to 4 were analysed by the first and third authors. In the second step, coding proceeded primarily from the data, for example “Language as a key to employment”, “Body language as a cultural expression”, “Emotional support from social networks”, “Starting supportive initiatives”, “Created a professional identity”, “Contrasting sensory cultures”, “Reclaiming agency across time” and “Strategic temporal action” (Table 2). However, the abductive stance allowed for attention to both expected and unexpected patterns. When unexpected elements emerged, they were explored in dialogue with the theory [27, 28], prompting refinements to the coding decisions. In the third step, the codes were discussed, compared, and organised based on similarities and differences. This analysis step yielded 16 tentative sub-themes. During the fourth step, the abductive logic supported a back-and-forth movement between coded extracts, tentative subthemes, and theoretical ideas. This recursive comparison ensured that the subthemes were grounded in the narratives while also capturing theoretically meaningful dimensions. The tentative subthemes were reviewed, discussed several times, and revised to reflect the experiences of immigrant women. This ended up with 8 subthemes. Finally, the fifth step concerned defining and naming themes. The final refinement of themes was informed by theory [27, 28], which helped articulate the broader significance of the empirical patterns. Theoretical insights were used to deepen the explanatory power of the themes. Four main themes were formulated, and the analysis continued until all authors agreed that the four themes were distinct and robust with respect to the aim and the interview transcripts. Quotations from different participants have been chosen to illustrate the findings. All quotations were translated from Swedish into English and adjusted for readability.

**Table 2:** Overview of the themes, subthemes and examples of codes

Themes	Subthemes	Codes
Navigating Language and Everyday Communication	Learning Swedish as a Gateway to Work	Language as a key to employment
	Adjusting Dress, Behaviour, and Body Language in Swedish Workplaces	Body language as a cultural expression
Building Supportive Relationships and Networks	Receiving Support from Individuals and Community Contacts	Emotional support from social networks
	Supporting Other Immigrant Women	Starting supportive initiatives
Experiencing Work through Practice and Emotion	Entering New Work Environments and Professional Roles	Created a professional identity
	Differences in Workplace cultures and Interactions	Contrasting sensory cultures
Pathways of Change and Personal Initiative	Moving from Dependency to Independence	Reclaiming agency across time
	Strategic Efforts to Enter Working Life	Strategic temporal action

### Ethical considerations

According to the Act [37] on ethical review of research involving humans, ethical approval is required for research involving the processing of personal data, including sensitive personal data. This study received ethical approval from the Ethical Review Board (registration number: 2021–03543). The study complied with the Swedish Research Council’s [38] ethical requirements for research, including informed consent, confidentiality, and utilisation. All participants were informed of the research project’s outline, the study’s aim, how the collected data material would be handled, and the confidential procedures. Before the interview, the women received oral and written information about the project, in Swedish. As mentioned earlier, their participation in the study was voluntary and could be discontinued at any time without providing a reason. The collected material was processed in accordance with the General Data Protection Regulation. The data were stored in a locked cabinet, coded with non-identifiable codes, and only the researchers had access to them.

## Results

Four themes, including sub-themes, appeared when the immigrant women described their experiences and reflections of the processes and aspects that influence them to enter working life; *Navigating language and everyday communication*, *Building supportive relationships and networks*,* Experiencing work through practice and emotion* and *Pathways of change and personal initiative* (Table 3).

**Table 3:** Research questions and themes

Research questions	Themes
RQ1: How do immigrant women in Sweden describe and experience their pathways into working life?	Navigating Language and Everyday Communication
	Building Supportive Relationships and Networks
	Pathways of Change and Personal Initiative
RQ2: How do immigrant women in Sweden describe experience their workplace?	Experiencing Work through Practice and and Emotion
	Pathways of Change and Personal Initiative

### Navigating language and everyday communication

#### Learning Swedish as a gateway to work

Learning Swedish was described as crucial for immigrant women’s ability to enter working life and participate in everyday society. Several women emphasised that passing Swedish for Immigrants (SFI) was a formal requirement, but they also noted that their local environment shaped their opportunities to learn the language. Living in a segregated area made it difficult to meet Swedish speakers:

“Where are all the Swedes? So, finding someone to talk to and practice the language was a bit difficult. Therefore, the first year was tough; I only wanted a job. But finally, I got a job, actually two jobs, I think, because of my Swedish.” (Informant 8)

For some, motivation to learn Swedish shifted over time. One woman initially refused to learn the language because she did not see her future in Sweden: *“I didn’t want to learn Swedish! I even refused to speak Swedish. So*,* I was closed off from learning a single word.”* (Informant 1)

As she entered working life, she realised how language skills shaped everyday interactions and fairness at work:

“My first job didn’t go well because I didn’t speak Swedish well… Who can go to the boss faster and tell what happened? If you don’t speak Swedish, it’s unfair. When you can’t articulate what you want and how you feel.” (Informant 1)

Many immigrant women described their multilingual backgrounds as assets; however, Swedish was consistently perceived as the language that opened doors. One woman called it the *“currency of participation*,*”* capturing how proficiency in Swedish enabled recognition, trust, and access to opportunities. Workplaces also became central arenas for learning Swedish. Being surrounded by Swedish-speaking colleagues assisted several women in gaining confidence: *“Before*,* I was afraid to speak Swedish*,* but at work I had no choice. And that’s when I really learned.”* (Informant 6)

Overall, the immigrant women’s stories showed that integration into working life was most successful when they were not only placed in jobs but given opportunities to speak, interact, and grow in environments where their voices mattered.

#### Adjusting dress, behaviour, and body language in Swedish workplaces

The immigrant women’s accounts of dress and body language reveal how they navigated the cultural expectations present in Swedish workplaces. Many described combining aspects of their home culture with local norms in ways that felt both comfortable and socially acceptable. For one woman, wearing the hijab was a central part of her identity, but she meant that this is not always a religious decision. At work, it signalled her boundaries, such as not hugging. When colleagues respected this, she felt safe and accepted. At the same time, she noted that some immigrant women removed the hijab to avoid discrimination or missed job opportunities.

Others described differences in everyday gestures and body language. Colleagues often greeted each other with hugs or informal warmth, while some women initially held back. Over time, through Women On Wednesdays, an organisation providing foreign women networking opportunities (WOW, 2024a), employment, and voluntary activities, several women described becoming more at ease with these behaviours: *“I sat with them [colleagues] every day*,* watched how they write emails and speak during meetings… I listened all the time and learned.”* (Informant 7)

For some, adapting also meant learning to interpret humour, tone, or indirect communication. One woman explained: *“At first*,* I didn’t understand when someone joked. I thought they were serious. Now I can laugh with them.”* (Informant 3)

These accounts highlight a gradual, embodied learning process in which women adjusted aspects of dress, gesture, and tone as they navigated how to remain authentic to themselves.

### Building supportive relationships and networks

#### Receiving support from individuals and community contacts

The immigrant women’s pathways into working life were strongly shaped by support from engaged individuals and their networks. Some used contacts to find their first jobs, while others met informal mentors who offered guidance, encouragement, and emotional support: *“You always need support or a guide for getting a job… Someone who can help you with a lot of things. That you can just say your feelings and how you feel was also very important to me.”* (Informant 10)

One woman described how an internship led to long-term employment, with staff helping her prepare CVs and acting as references: *“They helped me write my CV and personal letter… And I got excellent references from them.”* (Informant 10) Several women described how relationships with Swedish families, volunteers, or colleagues became significant sources of belonging: *“A Swedish family invited me to their house… We are still friends*,* and it helped me get a job.”* (Informant 11) These relationships frequently filled gaps that formal integration structures could not address, offering both emotional support and practical knowledge.

### Supporting other immigrant women

Many immigrant women emphasised the importance of helping others once they were more established. This support took various forms, including accompanying newcomers to WOW, organising study groups, and offering advice. One of the women said, *“We help people enter working life… The Welcome project helps migrant women in the beginning.”* (Informant 11) Another woman started a book circle for women wanting to improve their Swedish. Through this initiative, she introduced others to WOW: *“Through the book circle*,* I met Anna… She needed support both in finding friends and getting a job.”* (Informant 8)

Furthermore, a woman described the satisfaction of giving back: *“I want to stand on my own two feet because I got help… And it’s more fun to give when you’ve received it.”* (Informant 12) Supporting others strengthened their own sense of purpose and contributed to a growing feeling of inclusion and belonging in Swedish society.

### Experiencing work through practice and emotion

#### Entering new work environments and professional roles

Workplaces were described as key spaces where immigrant women rebuilt professional identities through practical tasks, interaction, and recognition. One trained nurse from Iran described a powerful moment during her internship: *“I cried the first time I took a blood sample… I had a label: ‘You can’t handle anything.’ But no*,* it went well.”* (Informant 12)

Receiving a job title that reflected her competence strengthened her sense of legitimacy in the workplace. Others described similar experiences of learning by doing, gradually taking on more responsibilities and developing trust in their skills: *“At first*,* I was shaking*,* but after a while*,* they trusted me more. And I trusted myself more*,* too.”* (Informant 2) These embodied experiences, including interacting with clients and performing tasks, were central in shaping their confidence and sense of belonging at work.

#### Differences in workplace cultures and interactions

The immigrant women often compared the emotional atmospheres of different workplaces. One woman noted stark contrasts: “Women were more supportive, more patient. Men said, ‘*Just do it yourself.’”* (Informant 8). Another expressed how recognition shaped her experience: *“It’s a dream job. I feel appreciated and trusted.”* (Informant 9)

Others described feeling invisible or undervalued in certain workplaces while experiencing warmth and encouragement in others: *“In one job*,* I felt invisible. In the other*,* they asked me how I was every morning.”* (Informant 3) Such comparisons reveal how the emotional climate, interaction style, and social tone impacted their job satisfaction and sense of security.

### Pathways of change and personal initiative

#### Moving from dependency to independence

The immigrant women’s narratives describe long, shifting journeys from dependency to increasing independence. Many explained how migration allowed them to rethink their values, ambitions, and sense of identity. One woman reflected: *“It is better to do it yourself because the human being will be stronger… No one shall tell me what I shall become!”* (Informant 1) Several women described WOW as a turning point that offered structure, community, and new opportunities: *“When I started at WOW*,* I began to feel better. I had something to do*,* people to talk to.”* (Informant 4) Participation in language activities, job preparation, and social events helped women transition from a state of “waiting” to active engagement in shaping their own futures.

#### Strategic efforts to enter working life

The immigrant women emphasised that entering the working life required persistence and strategic action. They described sending numerous applications, attending recruitment fairs, and seeking internships as stepping stones. One woman described her pursuit of a job at a prestigious company: *“I said I would work there someday. I applied many times… Then one day they called me*,* and last week I signed the contract.”* (Informant 3) Women often used internships to demonstrate their abilities: *“I knew if I could just get an internship*,* I could show them what I can do.”* (Informant 10) Overall, their stories portray belonging as a gradual, often challenging process shaped by determination, continuous learning, and moments of opportunity.

## Discussion

This study explored immigrant women’s pathways into working life in Sweden through the analytical lens of belonging. The findings challenge dominant narratives of integration as a linear, individualised, and outcome-oriented process. Instead, belonging appears as a dynamic, negotiated practice shaped through language, relationships, recognition and time. In line with May [28, 32], the analysis illustrates that belonging is not a fixed end-state of successful integration but a continually evolving process through which women navigate structural constraints and cultivate social participation. This study highlights how belonging operates both as a source of health and empowerment and as a mechanism of regulation within the institutional and cultural logics of Swedish integration. Empowerment is understood here as a process through which women gain agency, resources, and the ability to influence the conditions of their lives over time [39].

Furthermore, the narratives reveal that belonging is deeply entangled with power and conditionality. Language emerged as both a key to access and a site of exclusion. The women consistently described the process of learning Swedish as essential for employment and everyday participation, echoing Rydell et al. [40] metaphor of language as “a key to society.” Their stories demonstrate how linguistic resources shaped their capacity to negotiate belonging and establish themselves in workplace and community settings, aligning with previous research [19]. At the same time, the women exposed the moral hierarchies embedded in linguistic expectations. Fluency in Swedish not only signalled competence but positioned them as deserving of inclusion. Those who mastered the language could “go to the boss faster,” while others risked being overlooked. These accounts show how linguistic hierarchies structured access to power and mediated everyday negotiations of fairness and recognition, underscoring that inclusion cannot be reduced to formal language acquisition alone [6, 21, 41]. In this sense, Swedish proficiency acted as a “currency of participation,” consistent with May’s [28] notion of cultural belonging as constructed through shared language and norms. Although formal initiatives such as Swedish for Immigrants (SFI) presume that linguistic mastery is both necessary and sufficient over time, for social participation [42], the immigrant women’s accounts demonstrate that formal instruction was insufficient without the relational and embodied dimensions of language use that unfolded in everyday contexts [21, 41]. Their reflections may challenge logics of assimilation that place the burden of cultural belonging on individuals [28].

The immigrant women’s dress and body language were used both as their home culture and local norms, for example, wearing the hijab with pride. One study described that wearing a hijab is often the same as being religious [43], but this is not always the reason, according to this immigrant woman. Azam et al. [43] described that 73% of immigrants working as physicians (both male and female) wore a hijab or beard, and 38% reported discrimination at the workplace. This topic needs to change, and implementing policies that accommodate workplace needs is important. To feel safe at work, the results showed that some women chose not to hug, which is sometimes common in Swedish workplaces, and to be accepted for who you are and what you want is of importance for well-being at work [15]. Managers in workplaces must be aware of this to create a good work environment. To facilitate refugee women’s participation in the working life in Sweden, it is crucial to target different groups of immigrant women and address their distinct experiences, needs, and interests [26].

Relational experiences were central to the immigrant women’s sense of belonging and to their entry into working life. Consistent with earlier research showing that social networks facilitate both employment and psychological well-being [4, 12, 18, 19], the findings highlight that it was not access to networks per se but the quality of relational encounters that mattered. Trust, moral affirmation, and sustained interpersonal investment enabled the women to move from isolation toward participation. Several described these relationships as forming a “new family.” Civil society organisations were particularly significant, acting as compensatory structures where formal integration programmes offered limited personalised support [21, 25]. However, society’s health communication can inspire refugees to focus on their health as a tool for managing integration [11].

Importantly, belonging emerged through ongoing relational practices rather than through single encounters. Everyday interactions, such as being invited into a Swedish home, receiving help with job applications, or being offered references, were moments when social distance was bridged, and moral equality affirmed. These findings align with May’s [28] notion of relational belonging, which is grounded in the principles of recognition and reciprocity. The fact that some relationships “counted” more than others, particularly those with native-born Swedes or institutional gatekeepers, reveals how power mediates who can be recognised and on what terms. At the same time, relational inclusion carried implicit demands. Access to networks and support often required women to navigate and adapt to dominant cultural expectations. Adjustments in dress, tone, and bodily comportment were common strategies for aligning with workplace norms implicitly coded as Swedish. These accounts expose the conditional nature of belonging: inclusion depended on managing difference in ways that remained intelligible and acceptable to the majority. In this sense, belonging functioned simultaneously as an achievement and a demand, reinforcing May’s [28] emphasis on the normative dimension of inclusion.

The sensory dimension of belonging further underscores its embodied character. Several immigrant women described crying, shaking, or feeling physically relieved when recognised by supervisors or colleagues, experiences they interpreted as turning points in their integration. These affective reactions illustrate that belonging is not merely cognitive but corporeal and relational, echoing earlier findings that employment can restore self-esteem, social value, and emotional stability among refugees [7, 8]. Yet, the intensity and rarity of these moments also reveal the precariousness of their position: affirmation was not routine but contingent on being acknowledged by those in power. This aligns with research showing that refugee women often rely on sporadic recognition within hierarchical workplace structures, where inclusion must be repeatedly earned [12]. In this sense, sensory belonging becomes a site where power is felt through the body, exposing the intertwining of recognition and vulnerability. As such, the workplace functioned not only as a site of adaptation but also as an affective economy. Here, trust, embodied confidence, and professional identity circulated alongside formal markers of inclusion [15].

A temporal reading of the immigrant women’s narratives reveals how belonging unfolded over time as they moved from dependency to increasing independence, a trajectory reflected in their strategic efforts to enter the labour market. Their stories illustrate how they navigated prolonged periods of waiting, experiences that mirror broader migration time regimes marked by disrupted rhythms and uneven future orientations [44]. As the women reinterpreted their past lives while imagining new futures, belonging emerged not as a fixed state but as a temporal process shaped by shifting forms of agency. Moments of sudden momentum, often triggered by new relationships, opportunities, or routines, illustrate how temporal belonging took shape through shifts in direction rather than linear progression. This resonates with May’s [32] conceptualisation of belonging as a negotiation of continuity across past, present, and future selves. Civil society organisations, such as WOW, played an important role as temporal anchors, helping women re-establish daily rhythms, develop routines, and cultivate future-oriented aspirations, consistent with research showing that civic participation fosters agency and facilitates pathways to employment [18, 21, 22].

These temporal dynamics also shaped the immigrant women’s experience of integration. Their strategic efforts to gain access to education, training, and employment illustrate how integration is embedded in everyday negotiations of belonging, in which past resilience is mobilised to navigate present constraints and envision possible futures. This interpretation aligns with research demonstrating that participation in working life is closely intertwined with psychological well-being, belonging, and health, and that work constitutes a key arena in which belonging is either supported or constrained [4, 8, 25]. Importantly, a study of immigrant women in Sweden similarly emphasises that faster and more accessible pathways into work are crucial for both integration and well-being, underscoring the central role of timely opportunities in shaping women’s ability to transition from dependency to independence [45].

Overall, these findings move beyond structural or linguistic understandings of integration and conceptualise belonging as a transformative, socially embedded, and embodied process. Civil society organisations, workplaces, and community networks emerge as critical infrastructures where belonging is cultivated over time through recognition, relational investment, and affective engagement. Supporting such processes requires reframing integration policies away from narrow expectations of individual adaptation toward investments in relational, emotional, and embodied forms of participation. Recognising belonging as an evolving rather than an achieved state highlights the need for policies that accommodate uncertainty, foster connection, and enable refugee women to rebuild continuity, agency, and community in a new society. However, Sweden’s increasingly restrictive migration policies, particularly those affecting non-European migrants and refugees, might shape immigrant women’s pathways into the workforce [3].

### Strengths and limitations

To ensure trustworthiness, with particular emphasis on credibility, several strategies were employed in this study [46]. A strict and transparent analytical procedure, based on Braun and Clarke [35], was followed, contributing to methodological rigour and dependability. The research team’s extensive experience in qualitative methodology and immigration research supported a reflexive and well-informed analytical process, thereby strengthening the confirmability of the findings.

Credibility was further enhanced by the depth and richness of the data, which were supported by lengthy interviews that allowed participants to provide detailed narratives of their experiences. Sample size adequacy was guided by Malterud et al.’s [47] concept of *information power*. After 11 interviews, the information power was assessed as sufficient, given the richness and depth of the data and the inclusion of participants highly relevant to the study aim, who also represented variation in the experiences explored. Data collection was therefore concluded.

The use of both online and face-to-face interview modes broadened participation and increased inclusivity, particularly for women who were unable or unwilling to participate in person. Recruitment was facilitated by gatekeepers who helped establish trust, which is especially important when working with potentially vulnerable populations such as immigrant women.

Interviews were conducted with immigrant women who had direct experience of integration and the working life, ensuring that the data were rich and grounded in participants lived experiences.

Despite these strengths, several methodological limitations should be acknowledged. The involvement of gatekeepers may have shaped the sample in ways not fully visible to the researcher and could have affected who felt able or invited to participate. The dual interview modes introduced variability in the types and depths of data generated; for instance, online interviews may lack the richness of nonverbal communication. Although the interviews were conducted in a shared language, occasional linguistic barriers may have influenced the depth and clarity of participants’ narratives. Nuances, culturally embedded expressions, or emotionally charged descriptions may not always have been fully captured or articulated, potentially affecting both the richness of the data and the researcher’s interpretation. Additionally, the emotional intensity and uneven interview durations led to variability in narrative detail and posed challenges for data analysis. Finally, while the study provides some new insight into individual immigrant experiences, the limited sample size limits the extent to which the findings can be generalised beyond the specific participants.

## Conclusion and implications

Together, cultural, relational, sensory, and temporal aspects of belonging demonstrate that the immigrant women’s trajectories are characterised by both agency and constraint; while they strategically cultivated networks and opportunities, their ability to belong was shaped by institutional and cultural hierarchies. These findings challenge policy-oriented models that equate integration in working life with integration in society as a whole. Instead, they demonstrate that the ways immigrant women are received, recognised, and able to act within workplaces profoundly shape their sense of identity, agency, and social inclusion: *“I said I would work there someday. I applied many times… Then one day they called me*,* and last week I signed the contract.”* This is important to have in mind when new restricted policies are shaped by the government in Sweden.

Overall, the study contributes to understanding integration as a temporal and embodied process, in which belonging unfolds through immigrant women’s capacity to act, plan, and project themselves into meaningful futures. Their transition from dependency to independence thus reflects not only economic adjustment but a reconfiguration of selfhood and belonging over time.

Programs that facilitate mentorship, community-building, and skill development can be instrumental in helping women transition into professional environments. Furthermore, allowing immigrant women to engage in meaningful work early in their resettlement could promote a sense of belonging.

Future research would benefit from longitudinal designs to capture changes over time among immigrant women as they transition into pathways into working life.

## Supplementary Information

Supplementary Material 1.

## Data Availability

The datasets generated and analysed during the current study are not publicly available due to the sensitive nature of the data from a vulnerable group, and cannot be freely shared. However, data are available from the corresponding author upon reasonable request, provided that consent has been obtained from the research participants.
