# Supplementary material for: Belonging as a part of the pathway to working life: immigrant women’s experiences in Sweden
Source: BMC Public Health. 2026 Oct 5;26:2808. doi: 10.1186/s12889-026-29688-w (PMC13640177; doi:10.1186/s12889-026-29688-w)
Supplement: Supplementary file 1 [file 12889_2026_29688_MOESM1_ESM.docx]

**Interview guide working life**

**Introduction**

• Thank you very much for wanting to participate in the study!

• Brief introduction of the interviewer

• Brief introduction of the study

• Audio documentation (digital audio recording on a closed device)

• Ethical aspects (anonymity, cancel participation)

**Home country/past time**

• What did you do in your home country?

• Did you have any work in your home country? If so, what kind of work did you have?

• What were the conditions for young women to work in your home country?

**Sweden/present time**

• Describe how you experienced your first period in Sweden?

• Describe what you do today (with a focus on working life)? Follow-up questions based on the answers.

• Describe your current situation (with a focus on working life)?

• Describe how it went when you got your first job in Sweden?

• What has influenced you to get the job you have today (conditions, people, events, norms (your own or others))?

• What does it mean to you to have a job?

• What has been important for you to feel good in working life?

**Ending**

• Is there any question you would like to return to or anything you would like to add?

• Thank you very much for wanting to participate in the study!
